# Uterine Adenosarcoma: A Retrospective 12-Year Single-Center Study

**DOI:** 10.3389/fonc.2019.00237

**Published:** 2019-05-14

**Authors:** Zhen Yuan, Mei Yu, Keng Shen, Jiaxin Yang, Dongyan Cao, Ying Zhang, Huimei Zhou, Huanwen Wu

**Affiliations:** ^1^Department of Obstetrics and Gynecology, Peking Union Medical College Hospital, Peking Union Medical College, Chinese Academy of Medical Sciences, Beijing, China; ^2^Department of Pathology, Peking Union Medical College Hospital, Peking Union Medical College, Chinese Academy of Medical Sciences, Beijing, China

**Keywords:** uterine adenosarcoma, fertility-sparing surgery, lymphovascular space invasion, presence of tumor stalk, hysteroscopy, chemotherapy

## Abstract

**Synopsis:** Lymphovascular space invasion is an independent risk factor for disease progression and presence of tumor stalk an independent protective factor. Fertility sparing surgery may be acceptable in cases whose tumors present with stalks and without high risk factors.

**Objectives:** The aim of the present study was to investigate the potential prognostic factors of uterine adenosarcoma.

**Methods:** A total of 49 cases of uterine adenosarcoma were retrospectively reviewed at our institution between April 2006 and October 2018.

**Results:** Median follow-up time was 34 months (range: 1–148). Median age was 47.50 years (19–75). Nineteen (38.9%) patients were uterine cervical adenosarcoma and 30 (61.22%) patients were uterine corpus adenosarcoma. Twenty-nine (59.2%) patients were polypoid with a stalk to the uterine cervix or corpus. Twenty-six (38.8%) patients were stage IA. Fifteen (30.6%) patients showed sarcomatous overgrowth. Six (12.2%) patients displayed lymphovascular space invasion (LVSI). Four (8.16%) patients had heterologous elements. In univariate analysis, Disease-free-survival (DFS) was associated with tumor location, presence of tumor stalk, heterologous elements, LVSI. In multivariate analysis, presence of tumor stalk remained an independently protective factor for recurrence (HR = 0.088, *P* = 0.005), and LVSI a risk factor for recurrence (HR = 11.953, *P* = 0.002). Fertility-sparing surgery (FSS) was performed in seven stage IA patients. When patients of stage IA analyzed separately, FSS was not significant with the DFS or OS.

**Conclusions:** Presence of tumor stalk remained an independently protective factor for recurrence. Along with adequate counseling, FSS may be acceptable in cases whose tumors present with stalks and without high risk factors.

## Introduction

Mullerian adenosarcoma is a rare mixed tumor of low malignant potential that shows an intimate combination of benign glandular epithelium and low-grade sarcoma, usually of the endometrial stromal type (1). Clement and Scully provided the first description of uterine adenosarcoma in 1974 (2). Uterine adenosarcoma represents 5.5–9.0% of uterine sarcomas and ~1% of female genital tract malignancies (3). The treatment for uterine adenosarcoma is based on the limited available data for its rarity. The rarity of uterine adenosarcoma has made it difficult to examine a large patient population in a uniform manner and to collect reliable data concerning their prognosis. Comprehensive knowledge of prognosis based on clinicopathological features and treatment has important implications.

To investigate the potential prognostic factors of uterine adenosarcoma, we conducted a retrospective study including data of 49 cases that were treated in our institution, of which 19 cases were of cervix and 30 cases were of uterine body. Moreover, seven cases of stage IA were treated with fertility sparing surgery (FSS). As far as we know, our study is the largest study on fertility preservation in stage IA uterine adenosarcoma that has been performed at a single center.

## Materials and Methods

Our research was approved by the ethics committee of Peking Union Medical College Hospital (PUMCH), the need for written informed consent was waived due to the retrospective nature of the study, and all personal information has been de-identified to protect patient privacy.

### Patient Information

This study was based on the information of patients diagnosed and treated at PUMCH, between April 2006 and October 2018. Patient information, including age of onset, chief complaint, clinical features, treatment modality, and outcome of the treatment was collected from the medical records. The follow-up information was obtained from out-hospital medical records and via telephone. All of the follow-up information was censored following October 18, 2018.

### Pathological Review

With blinding to original diagnosis, all specimens were diagnosed randomly by two independent pathologists from the Department of Pathology at Peking Union Medical College Hospital. The cases were included in this study, only if the diagnosis from two pathologists are identical with each other. Pathological staging was performed in accordance with the International Federation of Gynecology and Obstetrics staging system (4).

### Statistical Analysis

Tumors that occurred only in the cervix were defined as uterine cervical adenosarcomas, and those that occurred in corpus with or without cervical infiltration were defined as uterine corpus adenosarcomas. Sarcomatous overgrowth was diagnosed when the pure sarcomatous portion of the neoplasm constituted more than 25% of the primary tumor (5). The duration of the patients' overall survival (OS) was calculated from the date of the initial surgery to the date of death or last contact, and their disease-free survival (DFS) was measured from the date of the initial surgery to the date of first progression or recurrence. Categorical variables are summarized in frequency tables, whereas continuous variables are presented as median (range). Frequency distributions were compared using chi-squared or Fisher' s exact tests and median values were compared using Mann-Whitney nonparametric *U* tests. The product-limit method of Kaplan and Meier was used to estimate OS and DFS, and the difference in survival between groups was tested using Log-rank test. Variables with *P* < 0.1 on univariate analysis were selected for multivariate analysis. Cox proportion hazards model was utilized for multivariate regression analysis of survival data. All statistics analysis was conducted using SPSS version 23 (IBM Corp, Armonk, NY, USA). A *p*-value < 0.05 was considered statistically significant with the two-tailed hypothesis.

## Results

### Clinical Characteristics

Forty-nine patients with uterine adenosarcoma was retrospectively identified. The demographics and characteristics of tumors are summarized in Supplementary Table 1. The median age at diagnosis was 47.50 years (range, 19–75 years). The diagnosis of 18 (36.7%) patients were established through hysteroscopy. Nineteen (38.78%) and thirty (61.2%) patients were diagnosed with uterine cervical adenosarcoma and uterine corpus adenosarcoma, respectively. The median body mass index (BMI) was 23.50 (range, 16.73–36.21). Twenty-nine (59.2%) patients presented with abnormal vaginal bleeding, 6 (12.2%) patients showed prolapse of mass through vaginal ostium, 1 (2.0%) patient showed an increasing of vaginal discharge, 2 (4.1%) patients had abdominal pain and the other patients were found by routine examination without complaint. Sixteen (32.7%) patients were nulliparous. Eight (16.3%) patients had dysmenorrhea. The median value of CA 125 preoperatively was 42.05 U/ML (range, 7–2,651 U/ML).

### Histological Characteristics

**Figure 1:**
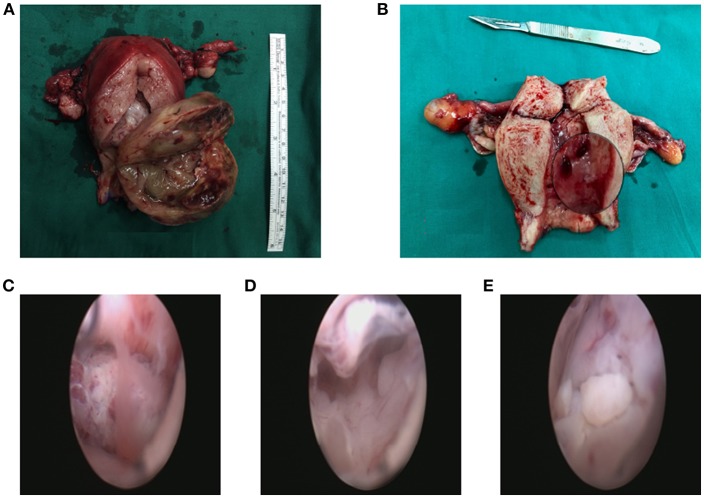
The tumor with a stalk to the uterine **(A)**. The residual stalk root after tumor resection **(B)**. Extensive lesions without a stalk **(C–E)**.

Most tumors (29 patients, 59.2%) were polypoid with a stalk to the uterine cervix or corpus. Twenty patients showed extensive tumor without obvious stalk. Figure 1 shows the tumors with and without stalks, respectively. The median maximum diameter of tumor size was 6 cm (range, 0.5–15 cm). According to the FIGO staging system, 26 patients (38.8%) patients were of stage IA, 13 (26.5%) patients IB, 2 (4.1%) patients IC staging, 2 (4.1%) patients IIA, 2 (4.1%) patients IIB, 1 (2.0%) patients IIIA staging and 3 (6.1%) patients IIIB. Fifteen (30.6%) patients showed sarcomatous overgrowth. Four (8.2%) patients had heterologous elements, of which 2 was with striated muscle sarcoma, 1 with epithelial cancer, 1 with sex cord tumor. Six (12.2%) patients displayed lymphovascular space invasion (LVSI) and 5 (10.2%) patients showed tumor necrosis. No pathological evidence of lymph node metastasis was found. In patients for whom immunohistochemical staining was performed, the positive rate of CD10 was 79.3% (23/29), P53 was 38.5% (5/13), estrogen receptor (ER) was 84.5% (22/26), progesterone receptor (PR) was 69.2% (18/26). The median value of Ki 67 was 20% (2–91%).

### Therapeutic Procedures

**Figure 2:**
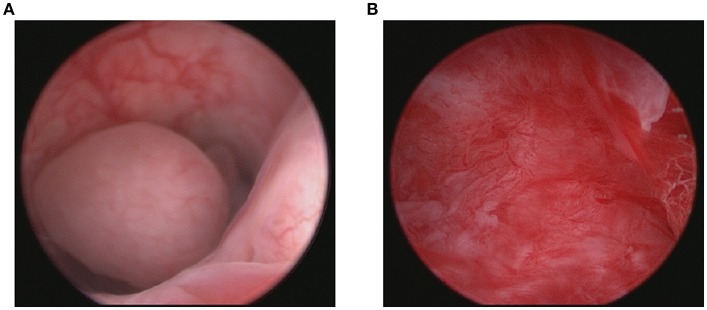
Tumor before resection **(A)** and the cavity after tumor resection **(B)** during the hysteroscopy.

FSS was performed in seven patients (Table 1) through hysteroscopy and tumor resection. Figure 2 shows the tumor before resection and the cavity after tumor resection during the hysteroscopy. The remaining 42 patients underwent total hysterectomy (TH). Of these patients, eight patients simultaneously underwent bilateral salpingectomy (BS), 24 patients bilateral salpingoopherectomy (BSO) and 10 patients staging surgery. Of those 10 patients, one patient underwent TH + BS + lymph node dissection (LND), three patients underwent TH + BSO + LND, three patients underwent TH + BSO + LND+ omentectomy (OMEN), and three patients underwent TH + BSO + OMEN + LND + appendicectomy.

**Table 1:** Clinical and histological characteristics of patients with fertility-sparing surgery.

**Case**	**Age (years)**	**Tumor size (cm)**	**Tumor location**	**Presence of tumor stalk**	**SO**	**HE**	**LVSI**	**FIGO stage**	**Surgery**	**Adjuvant therapy**	**Follow-up(m)**	**Fertility outcome**	**Oncological outcome**
1	27	2	Cervix and Uterine corpus	NO	NO	NO	NO	IA	Hys + TR	Chemo, PEI^*^3; Hormo, MPA;	148	-	Relapse twice; AWD;
2	19	6	Cervix	YES	YES	NO	NO	IA	Hys + TR	Chemo, PE^*^3	13	Infertility	NED
3	32	4	Cervix	YES	YES	NO	NO	IA	Hys + TR	Chemo, PE^*^2; Hormo, MA	10	Infertility	NED
4	34	3	Cervix	YES	NO	NO	NO	IA	Hys + TR	NO	57	Contraception	NED
5	31	3.5	Cervix	YES	NO	NO	NO	IA	Hys + TR	Hormo, GnRH-a	15	Contraception	NED
6	31	1	Uterine corpus	YES	YES	NO	NO	IA	Hys + TR	NO	59	Pregnancy 12 m after Hys	NED
7	29	1.5	Uterine corpus	YES	NO	NO	NO	IA	Hys + TR	Hormo, MA	27	Contraception	NED

*M, months; Hys, Hysteroscopy; TR, Tumor resection; chemo, chemotherapy; Hormo, hormonotherapy; AWD, alive with disease; NED, no evidence of disease; MPA, medroxyprogesterone acetate; MA, megestrol acetate*.

Of the total 49 patients, 12 patients received chemotherapy after the treatment surgery, including PEI (cisplatin+ epirubicin + ifosfamide), PE (cisplatin+ epirubicin), PI (cisplatin + ifosfamide), and others (gemcitabine+ docetaxel). The median course of chemotherapy was 3 (range, 2–8). Six patients received radiotherapy and nine patients received hormone therapy.

### Oncologic Outcomes

Follow up information was available for 48 patients. One patient died due to gastrointestinal malignant tumor 1 years after the initial surgery, without evidence of the uterine adenosarcoma spread, and was considered to be censored. The median follow-up time was 34 months (range, 1–148 months) for all patients. Totally 10 (20.4%) patients showed tumor progress, of whom one patient died 1 month after the surgery with rapid progression of disease and nine patients showed relapse. Local relapse occurred in eight patients and distant relapse (in the lung) in one patient. Of the nine recurrent cases, five patients died of uterine adenosarcoma and four patients are alive with disease. Thus, overall, the death rate was 6/49 (12.2%).

**Figure 3:**
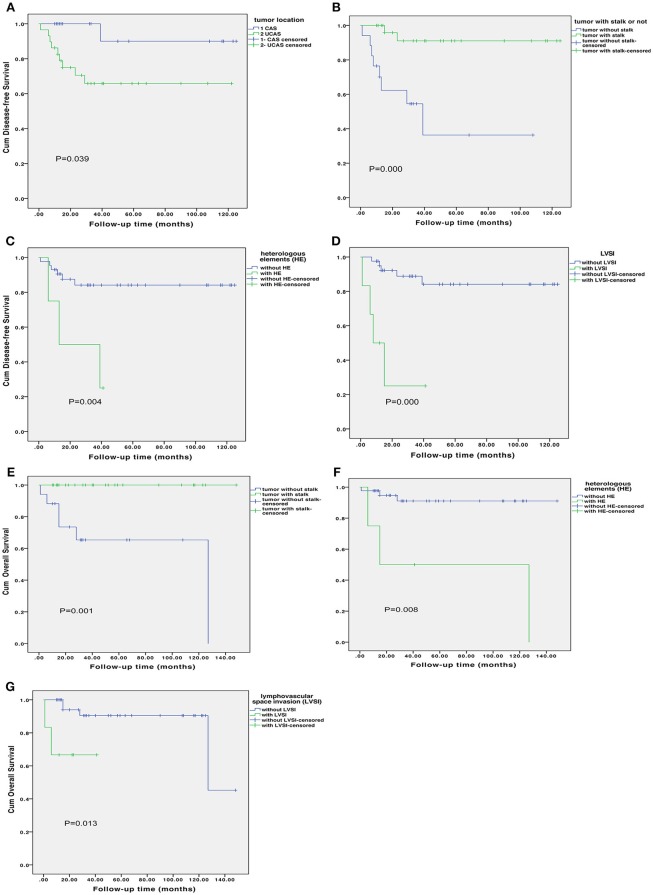
Disease-free survival according to tumor location **(A)**, presence of tumor stalk **(B)**, heterologous elements **(C)** and lymphovascular space invasion [LVSI; **(D)**]. Overall survival according to presence of tumor stalk **(E)**, heterologous elements **(F)** and lymphovascular space invasion **(G)**.

Supplementary Table 2 shows the risk factor for disease progress in uterine adenosarcoma. In univariate analysis, DFS was significantly associated with tumor location (*P* = 0.039; Figure 3), presence of tumor stalk (*P* < 0.000; Figure 3), heterologous elements (*P* = 0.012; Figure 3), LVSI (*P* < 0.000; Figure 3). Tumor size, FIGO stage, sarcomatous overgrowth and myometrial invasion were not statistically associated with DFS. In multivariate analysis, presence of tumor stalk remained an independent protective factor for recurrence (HR = 0.088, 95% CI = 0.016–0.482, *P* = 0.005), and LVSI remained an independent risk factor for recurrence (HR = 11.953, 95% CI = 2.482–57.580, *P* = 0.002). Supplementary Table 2 shows the risk factor for OS. In univariate analysis, presence of tumor stalk (*P* = 0.001; Figure 3), heterologous elements (*P* = 0.008; Figure 3) and LVSI (*P* = 0.013; Figure 3) were significantly associated with OS. In multivariate analysis, no statistically significant association was found.

With regard to FSS, when patients, who were of FIGO stage IA, were analyzed separately, FSS was not significantly associated with the DFS or OS (*P* = 0.396, *P* = 0.564, respectively; Supplementary Figure 1).

### Menstruation and Fertility

Seven patients with FSS were under close follow-up, with the median follow-up time being 27 months (rang, 10–148 months). Of these seven patients, one patient showed recurrence 23 months after the initial treatment. TH + BS and bilateral ovary biopsy were performed. Postoperative pathological diagnosis was uterine adenosarcoma with myometrial invasion. Pelvic recurrent tumor resection, bilateral oophorectomy and lymph node dissection, with no residual tumor, were performed 30 months after the first recurrent surgery. Radiotherapy was performed postoperatively. During the radiotherapy, multiple minor lesions were found in the lung without complaint. With close follow-up, no further treatment was given. After 74 months, parts of the lesions were noted to have become slightly bigger and biopsy was performed. The pathological diagnosis was metastatic low-grade uterine adenosarcoma, with strongly positive ER and PR. Letrozole was given and the patient was under close follow-up 7 months after the lung lesion biopsy. Until her latest follow-up, the follow-up time was about 148 months.

The remaining six patients showed normal menstruation, one of whom was taking oral contraceptives. The median follow-up time was 21 months (range, 10–59 months). Of these six patients, three tried to achieve a pregnancy. One of these three patients had experienced a full-term pregnancy, 12 months after the initial surgery, and the other two remained infertile after 10 months and 13 months, respectively.

## Discussion

In this study we reviewed the demographics, clinicopathological characteristics and oncologic results of 49 patients with adenosarcoma, and reported the largest series of FSS in stage IA uterine adenosarcoma.

As far as we know, the percentage of cervical adenosarcoma in uterus reported in our study is larger than that in previous reports (6, 7). One reason for this may be that the use of hysteroscopy accurately aided in identifying the location of tumor. In this study, the patients with uterine cervical adenosarcoma were younger than those with uterine corpus adenosarcoma, consistent with the results of a previous study (8).

Adenosarcomas usually presents as a soft polypoid mass (1). Our study confirmed this observation, with 59.18% of the adenosarcoma presenting polypoid with a stalk to the cervix or uterine corpus.

The majority of patients (73.4–82%) are diagnosed with stage I disease (3). In our study, 83% of the patients were FIGO stage I. TH and BSO were recommended for the majority of uterine adenosarcoma patients (9). In our study, 24 patients (48.97%) underwent TH and BSO. Aggressive surgery was performed for 10 patients and relative conservative surgery for 15 patients, of whom seven patients underwent hysteroscopy and tumor resection for fertility preservation. Regarding the adjuvant therapy, no overall survival benefit for radiotherapy has been noted (9, 10), and there may not be enough evidence of benefit for chemotherapy and hormonotherapy (3). In this study, 23 patients received adjuvant therapy, including chemotherapy, radiotherapy, and hormonotherapy. Results showed no difference in DFS and OS. As the previous study reported (9), in fact, there is a trend toward worse prognosis for patients undergoing adjuvant therapy, conceivably the result of selection bias, has the higher-risker patients may have been selected to undergo adjuvant. The current data, and prior reports have not shown efficacy of any adjuvant therapy, though limited by small sample size, variations in adjuvant treatment. And further research required to identify the most effective adjuvant treatments, and the patient population at highest risk of recurrence in which adjuvant therapy should be studied.

Previous studies reported that recurrences rate is 14.3–46% of patients with uterine adenosarcoma, with local recurrences being more common than distant recurrence (11, 12). In our study the recurrence rate is 18.37 and 88.89% being local recurrence, consistent with previous studies.

Another previous study has suggested sarcomatous overgrowth, myometrial invasion, size, mitosis, age, race, FIGO stage, resection status, necrosis, cellular atypia, heterologous elements, and rhabdomyosarcoma elements are possible prognostic factors of adenosarcoma (3). In our study, using univariate analysis, we confirmed the observation that heterologous elements and LVSI were significant prognostic factors in DFS and OS. Moreover, we found presence of tumor stalk was a significant factor that affected DFS and OS. This can be explained by the fact that tumor with stalk may be easier to be resected completely, with a lower possibility of myometrial invasion, earlier stage, smaller size tumor. Some previous studies have reported the importance of tumor resection status is an important prognostic factor (9, 13). Other previous studies have reported that tumor with pedunculated tumor and uninvolved stalks can be curative by local excision (1, 14). All these results supported our observation. Moreover, there was a trend toward worse DFS and OS in patients with sarcomatous overgrowth. More aggressive surgery and adjuvant therapy were performed in patients with sarcomatous overgrowth in this study, which may affect the analysis. Moreover, because of the limited number, it may be insufficient to detect the difference. However, in this study, LVSI and presence of stalk were still significant with DFS, but not with OS. This may be explained by the small sample size of deaths in our study.

Whether FSS can be utilized in adenosarcoma is controversial. Some authors claimed that FSS would not be a preferred approach for its high risk in recurrence (9). Alternatively, some authors have reported that local tumor excision may been curative in some cases (Table 2) (6, 15–24). In previous studies, local tumor resection was done in 20 cases, two cases of which were alive with disease and four cases of which relapsed. Moreover, none of 20 cases underwent disease-related death. In this study, FSS was performed in 7 patients. One patient (14.28%) who underwent recurrence, is alive with disease, with a follow-up time of 148 months. In univariate and multivariate analysis, FSS was not significantly associated with DFS and OS. When patients with FIGO stage IA were analyzed separately, FSS was still not significantly associated with the DFS or OS. What is surprising is that one patients underwent full-term pregnancy and delivered a healthy baby successfully. Different from the study of Lee et al. (16), who have reported seven patients with fertility preservation, two patients were IB stage, of whom one patient were always alive with disease and the remaining one recurred. All the patients with FSS was stage IA. As long as we know, this is the largest series of FSS being conducted in patients with stage IA adenosarcoma. Consistent with the results of previous studies (6), FSS may be an acceptable choice of treatment in cases where tumors present with stalk and do not have high risk factors. It should be noted that, for FSS, hysteroscopy was essential. Hysteroscopy can be utilized not only to evaluate the uterine canal and cavity, identifying tumor locations, but also to confirm the complete tumor resection. Another point to emphasize is that, consistent with the recommendations of previous studies (6, 11), patients with sarcomatous overgrowth, which has been identified as high risk factor for recurrence and death (9), should not be recommended for FSS. However, in this study, three patients with sarcomatous overgrowth, even after being consented adequately of the risks, still required fertility preservation and underwent FSS. These patients are being closely followed up.

**Table 2:** Review of literatures about fertility-sparing surgery in uterine adenosarcoma.

**References**	**Age (years)**	**SO**	**Location**	**Tumor size (cm)**	**Treatment**	**Adjuvant therapy**	**Oncologic outcome**	**Fertility outcome**
Goh et al. (15)	21	NO	corpus	3	Hys + TR	NO	Local recurrence occurred after 8 years and LH + BSO + LND was underwent. DFS 43 m	Conceived and delivered a healthy infant, 3 years after surgery.
Togami et al. (6)	17	NO	cervix	Unknow	CKC	NO	NED 62 m	
Lee et al. (16)	33	NO	Unknown	Unknown	Hys + TR	NO	DFS	
	33	NO	Unknown	Unknown	Hys + TR	MPA	DFS	Conceived and delivered a healthy infant after surgery
	40	NO	Unknown	Unknown	D/C/Bx	NO	DFS	
	21	NO	Unknown	Unknown	Hys + TR	NO	AWD 17 m	
	22	NO	Unknown	Unknown	TR	NO	AWD 38 m	
	27	YES	Unknown	Unknown	Hys + TR	Chemotherapy	Recurrence after 13 m and TH + BSO was underwent. AWD 22 m	
	27	NO	Unknown	Unknown	Hys + TR	NO	Recurrence after 27 m. AWD 38 m	
Shinnick et al. (17)	14	No	cervix	4	CKC	No	Unknown	
Kanayama et al. (18)	28	No	cervix	5	CKC	no	Recurrence 3 m later and underwent TCR and endometrial curettage. DFS 32 m	Conceived naturally and delivered a healthy infant, 18 months after surgery
Sanamandra et al. (19)	13	No	cervix	4.2	TR	No	Unknown	
Chin et al. (20)	17	No but with HE	cervix	Unknown	CWR	No	DFS 204 m	Conceived and delivered a healthy infant, 9 years after surgery
Buyukkurt et al. (21)	14	Unknown	cervix	6^*^4	TR	no	DFS 15 m	
Geisler et al. (22)	23	No	cervix	3.5	Trache	No	Unknown	
Jones and Lefkowitz (23)	35	No	cervix	3.5	LE	no	DFS 120 m	
	35	No	cervix	Unknown	LE	no	Lost to follow	
	27	No	cervix	2	LE	no	DFS 12 m	
Zaloudek and Norris (24)	15	Unknown	cervix	Unknown	LE	no	DFS 48 m	Conceived and delivered a healthy infant
	15	Unknown	cervix	Unknown	Trache	no	DFS 72 m	

*SO, sarcomatous overgrowth; HE, heterologous elements; Hys, hysteroscopic; TR, tumor resection; LH + BSO + LND, laparoscopic total hysterectomy, bilateral salpingo-oopherectomy with bilateral pelvic lymph node dissection; D/C/Bx dilatation and curettage biopsy; CKC, cold knife conization; CWR, cervical wedge resection; LE, local excision; Trache, trachelectomy; TCR, transcervical resection; DFS, disease-free survival; M, months; Unknown, not mentioned*.

This study was limited by the inadequate large sample size and its retrospective nature, which could have possibly introduced some degree of bias. Despite these limitations, our study observed several important factors. The primary finding was regarding the prognostic factors for DFS. Presence of tumor stalk was the protective factor of recurrence. The second important finding was regarding FSS in patients with adenosarcoma.

In conclusion, our results confirm that LVSI presents a high risk for recurrence, and found that presence of tumor stalk remained an independently protective factor for recurrence. Along with adequate counseling, FSS may be acceptable in cases whose tumors present with stalks and without high risk factors. Patients should be counseled on risk of late recurrence. Close follow up should be mandatorily carried out.

## Author Contributions

ZY, KS, and MY conceived and designed the project. DC, JY, KS, and YZ collected the patients' characteristic data. HZ and HW prepared the figures and tables. ZY, MY, and DC analyzed and interpreted the data. ZY and MY wrote the manuscript. MY and KS are the corresponding authors. All the authors read and approved the final manuscript.

### Conflict of Interest Statement

The authors declare that the research was conducted in the absence of any commercial or financial relationships that could be construed as a potential conflict of interest.

## Supplementary Material

Supplementary Figure 1 Disease-free survival according to Fertility-sparing surgery (FSS) in stage IA patients **(A)**. Overall survival according to FSS in stage IA patients **(B)**.

Supplementary Table 1 Demographics and tumor characteristics of patients in our institution.

Supplementary Table 2 The risk factor for disease progression and death in univariate and multivariate analysis.

## References

[1] Clement PB Scully RE. Mullerian adenosarcoma of the uterus: a clinicopathologic analysis of 100 cases with a review of the literature. Hum Pathol. (1990) 21:363–81. 10.1016/0046-8177(90)90198-E 2156771

[2] Clement PB Scully RE. Mullerian adenosarcoma of the uterus. A clinicopathologic analysis of ten cases of a distinctive type of mullerian mixed tumor. Cancer. (1974) 34:1138–49. 10.1002/1097-0142(197410)34:4<1138::AID-CNCR2820340425>3.0.CO;2-9 4371193

[3] Nathenson MJ Ravi V Fleming N Wang WL Conley A Uterine adenosarcoma: a review. Curr Oncol Rep. (2016) 18:68 10.1007/s11912-016-0552-7 27718181

[4] Prat J. FIGO staging for uterine sarcomas. Int J Gynaecol Obstet. (2009) 104:177–78. 10.1016/j.ijgo.2008.12.008 19135669

[5] Seagle B-LL Falter KJ Lee SJ Frimer M Samuelson R Shahabi S Mullerian adenosarcoma of the cervix: Report of two large tumors with sarcomatous overgrowth or heterologous elements. Gynecol Oncol Case Rep. (2014) 9:7–10. 10.1016/j.gynor.2014.04.005 25426405 PMC4241484

[6] Togami S Kawamura T Fukuda M Yanazume S Kamio M Kobayashi H. Clinical management of uterine cervical Mullerian adenosarcoma: a clinicopathological study of six cases and review of the literature. Taiwan J Obstet Gynecol. (2018) 57:479–82. 10.1016/j.tjog.2018.04.032 30122564

[7] Tate K Watanabe R Yoshida H Shimizu H Uehara T Ishikawa M Uterine adenosarcoma in Japan: clinicopathologic features, diagnosis and management. Asia Pac J Clin Oncol. (2018) 14:318–25. 10.1111/ajco.12859 29441675

[8] Seagle BL Kanis M Strohl AE Shahabi S. Survival of women with Mullerian adenosarcoma: a National Cancer Data Base study. Gynecol Oncol. (2016) 143:636–41. 10.1016/j.ygyno.2016.10.013 27771166

[9] Nathenson MJ Conley AP Lin H Fleming N Lazar A Wang WL. The importance of lymphovascular invasion in uterine adenosarcomas: analysis of clinical, prognostic, and treatment outcomes. Int J Gynecol Cancer. (2018) 28:1297–310. 10.1097/IGC.0000000000001306 30044322

[10] Tanner EJ Toussaint T Leitao MM Jr Hensley ML Soslow RA Gardner GJ. Management of uterine adenosarcomas with and without sarcomatous overgrowth. Gynecol Oncol. (2013) 129:140–4. 10.1016/j.ygyno.2012.12.036 23283300

[11] Carroll A Ramirez PT Westin SN Soliman PT Munsell MF Nick AM. Uterine adenosarcoma: an analysis on management, outcomes, and risk factors for recurrence. Gynecol Oncol. (2014) 135:455–61. 10.1016/j.ygyno.2014.10.022 25449308 PMC4430193

[12] Benito V Lubrano A Arencibia O Andújar M Alvarez E Medina N. Clinicopathologic analysis of uterine sarcomas from a single institution in the Canary Islands. Int J Gynaecol Obstet. (2009) 107:44–9. 10.1016/j.ijgo.2009.05.020 19555952

[13] Machida H Nathenson MJ Takiuchi T Adams CL Garcia-Sayre J Matsuo K. Significance of lymph node metastasis on survival of women with uterine adenosarcoma. Gynecol Oncol. (2017) 144:524–30. 10.1016/j.ygyno.2017.01.012 28109626 PMC7523237

[14] Chen KT. Rhabdomyosarcomatous uterine adenosarcoma. Int J Gynecol Pathol. (1985) 4:146–52. 10.1097/00004347-198506000-00006 2991151

[15] Goh C Lin XH Chin PS Lim YK. Uterine preservation in a young patient with adenosarcoma of the uterus - Case report and review of literature. Gynecol Oncol Rep. (2018) 25:27–9. 10.1016/j.gore.2018.05.002 29977987 PMC6030025

[16] Lee YJ Kim DY Suh DS Kim JH Kim YM Kim YT. Feasibility of uterine preservation in the management of early-stage uterine adenosarcomas: a single institute experience. World J Surg Oncol. (2017) 15:87. 10.1186/s12957-017-1137-0 28424089 PMC5395796

[17] Shinnick JK Kumar N Beffa L Miller K Friedman MA Kalife E. Management of low-grade cervical Mullerian adenosarcoma in a 14-year-old girl. J Pediatr Adolesc Gynecol. (2017) 30:652–4. 10.1016/j.jpag.2017.05.010 28578185

[18] Kanayama S Nakamura M Oi H Sugimoto S Sasaki Y Uchiyama T. Case report of successful childbearing after conservative surgery for cervical Mullerian adenosarcoma. Case Rep Obstet Gynecol. (2017) 2017:4187416. 10.1155/2017/4187416 28154764 PMC5244008

[19] Sanamandra SK Leong MY Fortier MV. Vaginal mass in a 13-year-old girl. Ann Acad Med Singapore. (2014) 43:127–9. 24652436

[20] Chin PS Chia YN Lim YK Yam KL. Diagnosis and management of Mullerian adenosarcoma of the uterine cervix. Int J Gynecol Obstetr. (2013) 121:229–32. 10.1016/j.ijgo.2012.12.015 23490428

[21] Buyukkurt S Guzel AB Gumurdulu D Vardar MA Zeren H Sucu M. Mullerian adenosarcoma of the uterine cervix in an adolescent girl. J Pediatr Adolesc Gynecol. (2010) 23:e13–5. 10.1016/j.jpag.2009.05.008 19643645

[22] Geisler JP Orr CJ Manahan KJ. Robotically assisted total laparoscopic radical trachelectomy for fertility sparing in stage IB1 adenosarcoma of the cervix. J Laparoendosc Adv Surg Tech A. (2008) 18:727–9. 10.1089/lap.2007.0236 18803518

[23] Jones MW Lefkowitz M. Adenosarcoma of the uterine cervix: a clinicopathological study of 12 cases. Int J Gynecol Pathol. (1995) 14:223–9. 10.1097/00004347-199507000-00005 8600073

[24] Zaloudek CJ Norris HJ. Adenofibroma and adenosarcoma of the uterus: a clinicopathologic study of 35 cases. Cancer. (1981) 48:354–66. 10.1002/1097-0142(19810715)48:2<354::AID-CNCR2820480222>3.0.CO;2-Q 6263458

