# Corrigendum: Uterine Adenosarcoma: A Retrospective 12-Year Single-Center Study

**DOI:** 10.3389/fonc.2019.00657

**Published:** 2019-07-24

**Authors:** Zhen Yuan, Mei Yu, Keng Shen, Jiaxin Yang, Dongyan Cao, Ying Zhang, Huimei Zhou, Huanwen Wu

**Affiliations:** ^1^Department of Obstetrics and Gynecology, Peking Union Medical College Hospital, Peking Union Medical College, Chinese Academy of Medical Sciences, Beijing, China; ^2^Department of Pathology, Peking Union Medical College Hospital, Peking Union Medical College, Chinese Academy of Medical Sciences, Beijing, China

**Keywords:** uterine adenosarcoma, fertility-sparing surgery, lymphovascular space invasion, presence of tumor stalk, hysteroscopy, chemotherapy

In the published article, there was an error in the order of the authors. The second author should be “Mei Yu”. The correct order of the authors is “Zhen Yuan, Mei Yu, Keng Shen, Jiaxin Yang, Dongyan Cao, Ying Zhang, Huimei Zhou and Huanwen Wu”.

Furthermore, there was an error in the order of the corresponding authors. “Mei Yu” should the first corresponding author. The correct order of the corresponding authors is “Mei Yu, mypumc2000@126.com; Keng Shen, shenkengpumch@163.com”.

The authors apologize for these errors and state that this does not change the scientific conclusions of the article in any way. The original article has been updated.

