# Supplementary figures and images for: Uterine Adenosarcoma: A Retrospective 12-Year Single-Center Study
Source: Front Oncol. 2019 May 14;9:237. doi: 10.3389/fonc.2019.00237 (PMC6527837; doi:10.3389/fonc.2019.00237)

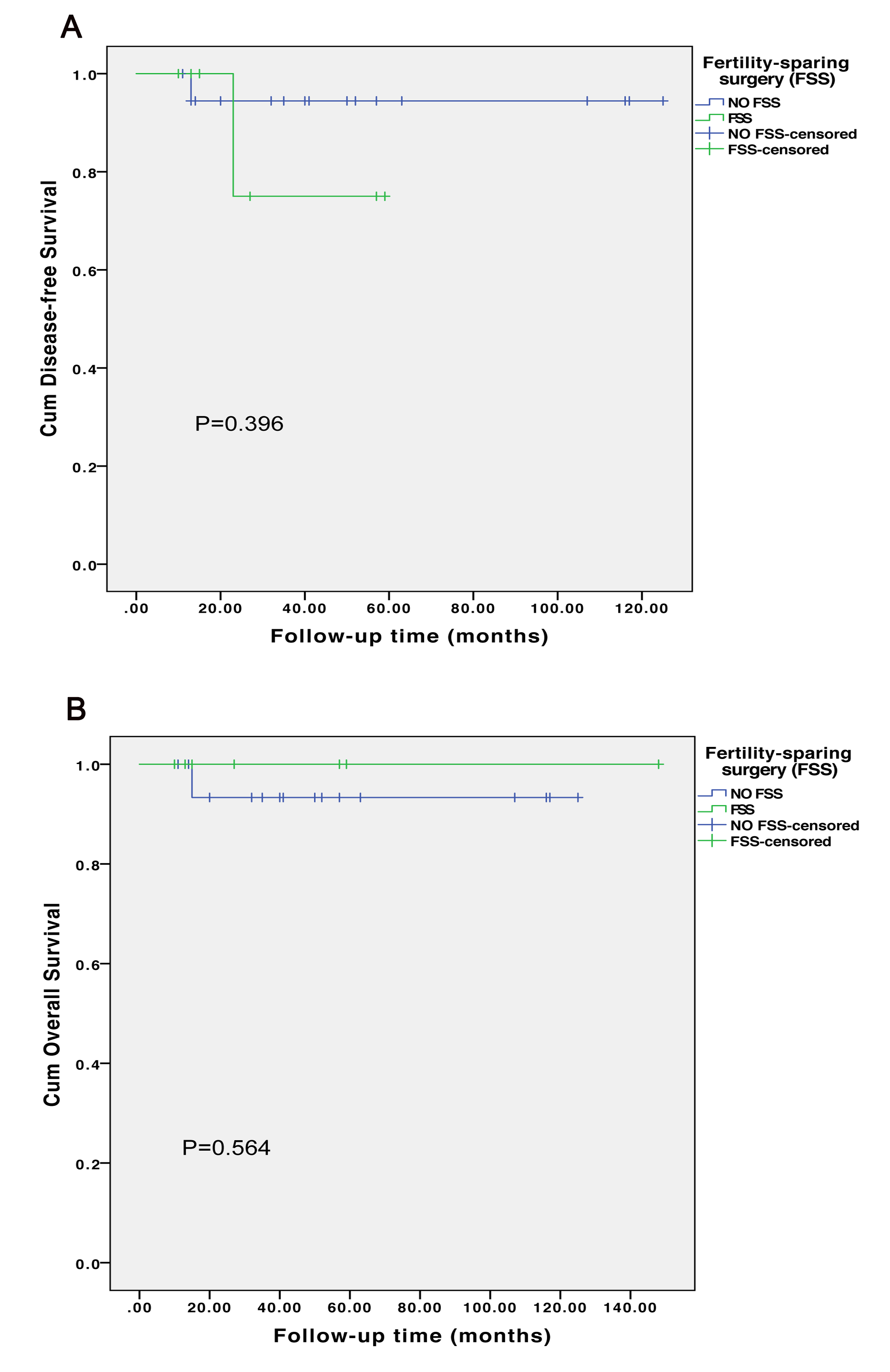

Supplement: Supplementary Figure 1 — Disease-free survival according to Fertility-sparing surgery (FSS) in stage IA patients (A). Overall survival according to FSS in stage IA patients (B). [file Image_1.TIF]
