# Supplementary material for: Uterine Adenosarcoma: A Retrospective 12-Year Single-Center Study
Source: Front Oncol. 2019 May 14;9:237. doi: 10.3389/fonc.2019.00237 (PMC6527837; doi:10.3389/fonc.2019.00237)
Supplement: Supplementary Table 1 — Demographics and tumor characteristics of patients in our institution. [file Data_Sheet_1.docx]

Supplementary table 1. Demographics and tumor characteristics of patients in our institution

| characteristics | n=49 |
| --- | --- |
| Age(year) | 47.50(19-75) |
| Nulliparous | 16(32.65%) |
| BMI (kg/m^2^) | 23.50  (16.73-36.21) |
| Dysmenorrhea | 8(16.32%) |
| CA125 (IU/L) | 40.05  (7.00-2651.00) |
| Tumor size (cm) | 6  (0.50-15.00) |
| Tumor with stalk | 29(59.18%) |
| FIGO Stage |  |
| IA | 26(53.06%) |
| Sarcomatous overgrowth; | 15(30.61%) |
| Heterologous elements | 4(8.16%) |
| Hymphovascular space invasion | 6(12.24%) |
| Fertility sparing surgery | 7(14.29%) |
| Disease progress rate | 10(20.40%) |
| Death rate | 6(12.24%） |
| Follow up(months) | 34.00  （1.00-148.00） |

BMI, body mass index; FIGO, International Federation of Gynecology and Obstetrics.
