# Supplementary material for: Uterine Adenosarcoma: A Retrospective 12-Year Single-Center Study
Source: Front Oncol. 2019 May 14;9:237. doi: 10.3389/fonc.2019.00237 (PMC6527837; doi:10.3389/fonc.2019.00237)
Supplement: Supplementary Table 2 — The risk factor for disease progression and death in univariate and multivariate analysis. [file Data_Sheet_2.docx]

Table 2. The risk factor for disease progression and death in univariate and multivariate analysis.

|  | Disease progression | | | Death | | |
| --- | --- | --- | --- | --- | --- | --- |
| Factors | Univariate | Multivariate | | Univariate | Multivariate | |
|  | P | HR (95% CI) | P | P | HR (95% CI) | P |
| Presence of tumor stalk |  |  |  |  |  |  |
| NO |  | 1 |  |  | 1 |  |
| YES | 0.000 | 0.088  （0.016-0.482） | 0.005 | 0.001 | 0.06  （0.000-9.421） | 0.175 |
| Tumor location |  |  |  |  |  |  |
| Cervical canal |  | 1 |  |  |  |  |
| Others | 0.039 | 5.403  (0.453-64.432) | 0.182 | 0.234 |  |  |
| FIGO stage |  |  |  |  |  |  |
| IA |  |  |  |  |  |  |
| Others | 0.194 |  |  | 0.128 |  |  |
| Tumor size(cm) |  |  |  |  |  |  |
| ≤5 |  |  |  |  |  |  |
| >5 | 0.456 |  |  | 0.635 |  |  |
| SO |  |  |  |  |  |  |
| NO |  | 1 |  |  | 1 |  |
| YES | 0.066 | 2.063  (0.384-11.075) | 0.398 | 0.061 | 2.035  (0.241-17.160) | 0.236 |
| HE |  |  |  |  |  |  |
| NO |  |  |  |  | 1 |  |
| YES | 0.004 | 2.783  (0.437-17.717) | 0.279 | 0.008 | 2.474  (0.332-18.447) | 0.178 |
| Myometrial invasion |  |  |  |  |  |  |
| NO |  |  |  |  |  |  |
| YES | 0.097 |  |  | 0.184 |  |  |
| LVSI |  |  |  |  |  |  |
| NO |  | 1 |  |  | 1 |  |
| YES | 0.000 | 11.953  （2.482-57.580） | 0.002 | 0.013 | 6.344  （0.680-59.176） | 0.055 |
| Surgical procedure |  |  |  |  |  |  |
| hysterectomy |  |  |  |  |  |  |
| FSS | 0.733 |  |  | 0.197 |  |  |
| Adjuvant therapy |  |  |  |  |  |  |
| NO |  |  |  |  |  |  |
| YES | 0.793 |  |  | 0.465 |  |  |

SO, sarcomatou overgrowth; HE, Heterologous elements; LVSI, Lymphovascular space invasion; FSS, Fertility-sparing surgery
